# Beverage‐Induced Discoloration and Surface Degradation of Single‐Shade Composite Resin: An In Vitro Investigation

**DOI:** 10.1155/ijod/3324637

**Published:** 2026-07-07

**Authors:** Ameesha Rai, Mythri Padaru, Disha Gupta, Raksha Bhat, Preethesh Shetty

**Affiliations:** ^1^ Department of Conservative Dentistry and Endodontics, AB Shetty Memorial Institute of Dental Sciences (ABSMIDS), Nitte (Deemed to be University), Mangalore, Karnataka, 575018, India, nitte.edu.in

**Keywords:** beverage staining, color stability, in vitro study, optical profilometry, single-shade composite resin, surface roughness

## Abstract

**Background:**

The longevity of direct composite resin restorations depends critically upon maintaining color stability and surface integrity throughout their clinical service life. Single‐shade composite resins, which utilize optical blending technology rather than conventional pigmentation, have been introduced to streamline restorative procedures. However, limited evidence exists regarding their susceptibility to extrinsic staining and surface degradation when exposed to acidic and chromogenic beverages commonly consumed in modern diets.

**Objective:**

This study aimed to evaluate and compare the effects of commonly consumed acidic and chromogenic beverages on the color stability and surface roughness of a single‐shade composite resin using spectrophotometric analysis and three‐dimensional optical profilometry.

**Methods:**

Forty disc‐shaped single‐shade composite resin specimens were made and divided into four groups (*n* = 10) at random: artificial saliva, kombucha, apple cider, and white wine. Specimens underwent daily 24‐h immersion cycles at 37°C in their respective solutions. Commission Internationale de l’Éclairage (CIE) LAB was used to measure color coordinates (*L* ^∗^, *a* ^∗^, and *b* ^∗^) colorimetry at baseline, 7 days, and 14 days. Surface roughness (Sp, Sv, Sz, and surface skewness [Ssk]) was assessed using three‐dimensional optical profilometry at baseline and 28 days. Statistical analysis employed within‐group comparisons using paired *t*‐tests and one‐way analysis of variance (ANOVA) with Tukey post hoc testing (*α* = 0.05).

**Results:**

In comparison to all other groups, kombucha significantly increased color change (Δ*E*) at 7 days (*p* < 0.001). By 14 days, all three test beverages produced statistically significant discoloration relative to artificial saliva, with kombucha demonstrating the highest Δ*E* values (*p* < 0.001). Analysis of CIE LAB coordinates revealed predominantly positive *Δ* *b* ^∗^ values in all beverage groups, indicating yellow‐ward discoloration. White wine produced the most pronounced increase in surface roughness parameters at 28 days (*p* = 0.016), followed by apple cider, while kombucha showed intermediate roughness alterations despite the greatest color change.

**Conclusions:**

Under the conditions of this investigation, single‐shade composite resin exhibits measurable and clinically significant changes in both color stability and surface topography following exposure to acidic and chromogenic beverages. The dissociation between staining severity and surface roughness alteration suggests distinct degradation mechanisms. These results highlight the need for dietary counseling and preventive measures to maximize the long‐term functional and esthetic performance of single‐shade composite restorations, which has significant implications for clinical practice.

## 1. Introduction

The esthetic and functional success of direct composite resin restorations is fundamentally dependent upon the substance’s capacity to maintain color stability and surface integrity throughout its clinical lifespan [1]. Composite resins have become the restorative material of choice in contemporary esthetic dentistry due to their favorable optical properties, conservative tooth preparation requirements, and direct placement technique [2]. However, these materials are inevitably exposed to a complex oral environment characterized by mechanical stresses, thermal fluctuations, water sorption, biofilm accumulation, and dietary chromogenic and acidic challenges [3, 4].

Composite resin discoloration represents one of the primary reasons for restoration replacement in clinical practice and represents a significant contributor to restoration failure [5]. This discoloration could result from material‐related intrinsic causes like composition, degree of polymerization, and resin matrix chemistry—or through extrinsic mechanisms involving the absorption or adsorption of pigments from external dietary sources [6]. Among extrinsic factors, acidic and chromogenic beverages have been extensively documented to compromise the resin composite material color and surface characteristics [7, 8].

Single‐shade (monochromatic) composite resins have been developed and adopted in recent years to reduce chairside time and eliminate the need for shade selection in clinical operations [9, 10]. Unlike conventional multishade composites that depend upon added pigments to achieve a specific shade, single‐shade composites rely on innovative optical properties including translucency, refractive index matching, and advanced filler technology to achieve a “chameleon effect” that permits the restoration to match the surrounding tooth structure in a variety of natural tooth colors [11, 12]. Despite these clinical advantages, concerns persist regarding the long‐term stability of these materials when subjected to extrinsic staining challenges. Furthermore, limited peer‐reviewed evidence exists contrasting the resilience of traditional multishade materials with single‐shade composites in response to beverage exposure.

Water sorption and resin matrix degradation play critical roles in the deterioration of composite resin properties. Hydrolytic breakdown of the polymer matrix weakens intermolecular bonding between polymer chains, leading to matrix softening, increased filler‐matrix debonding, and enhanced susceptibility to pigment penetration [13, 14]. The chemical composition of immersion media—including pH, titratable acidity, alcohol content, and presence of polyphenolic compounds—significantly influences the extent of both color change and surface degradation [15, 16].

Among beverages consumed in contemporary diets, several have not been comprehensively investigated for their effects on single‐shade composites. Kombucha, a fermented tea‐based beverage with increasing global consumption, contains organic acids, tannins, and polyphenolic compounds that may contribute to both staining and surface erosion. White wine, despite its lower chromogenic potential compared to red wine, contains ethanol and organic acids (tartaric, malic, and citric acids) that can accelerate resin matrix degradation and surface erosion. Apple cider, though relatively light in color, contains malic acid and natural pigments that may facilitate pigment adsorption into composite surfaces [17–19].

Surface roughness is an important parameter influencing both the esthetic and biological outcomes of composite resin restorations. Increased surface irregularities alter light reflection and optical properties, reduce gloss, and promote plaque accumulation and bacterial colonization [20, 21]. According to reports, staining sensitivity and biofilm retention significantly rise above a crucial surface roughness threshold of roughly 0.2 µm [22–24].

Three‐dimensional optical profilometry provides a noncontact method for detailed surface characterization without specimen damage, enabling comprehensive evaluation of surface topography beyond conventional linear roughness parameters [25, 26]. This technique permits simultaneous assessment of multiple ISO 25178‐defined parameters including peak height (Sp), total height (Sz), valley depth (Sv), and surface skewness (Ssk), providing greater insight into surface morphology than traditional Ra measurements alone.

The goal of the current study was to evaluate the color stability and surface roughness changes of a single‐shade composite resin (Omnichroma) following immersion in three beverages that reflect modern consumption patterns: white wine, apple cider, and kombucha, in contrast to a control of artificial saliva. These beverages were selected as they represent commonly consumed drinks in modern dietary patterns and exhibit diverse chemical characteristics, including variations in acidity, organic acid composition, alcohol content, and chromogenic compounds, making them relevant for evaluating their effects on composite resin properties. The research hypotheses were that (1) color change would be significantly greater in all beverage‐immersed specimens compared to controls, (2) kombucha would produce the greatest discoloration due to its high polyphenol and tannin content, and (3) white wine would induce the greatest surface roughness changes due to its ethanol and acid content.

## 2. Materials and Methods

### 2.1. Specimen Preparation

For this study, a single‐shade composite resin, Omnichroma (Tokuyama Dental, Tokyo, Japan), was selected as it represents a widely used material incorporating advanced structural color technology and supra‐nano spherical filler systems, making it suitable for evaluating the optical and surface behavior of contemporary single‐shade composites. Forty disc‐shaped specimens with standardized polyvinyl chloride (PVC) mold matrices were used to create dimensions of 6 mm diameter × 2 mm thickness. Disc‐shaped specimens were selected as they provide standardized geometry, uniform light‐curing conditions, and reproducible surface characteristics, which are critical for reliable comparison in in vitro dental material research. Although such specimens do not fully replicate the anatomical complexity of clinical restorations, they are widely accepted for controlled evaluation of material properties. The composite material was placed within the mold cavity between glass slides and Mylar strips to provide standardized and level surfaces. Light polymerization was carried out in accordance with the manufacturer’s instructions using an LED curing unit (Woodpecker; Guilin Woodpecker Medical Instrument Co., Guilin, China) with an output intensity of 1000 mW/cm^2^ for 30 s. The curing intensity was verified prior to specimen preparation, and the light‐curing tip was positioned perpendicular and in direct contact with the specimen surface to ensure uniform and standardized polymerization across all samples.

The Sof‐Lex Diamond Spiral Composite Finishing and Polishing System (3M ESPE, St. Paul, MN, USA) was used to finish and polish each specimen in accordance with the manufacturer’s instructions. In summary, this process produced a standardized, clinically representative surface finish by sequentially polishing utilizing medium, fine, and extra‐fine grits. This sequential polishing procedure simulates commonly employed clinical finishing and polishing protocols and ensures a standardized, clinically relevant surface finish across all specimens, which is critical for evaluating staining susceptibility. Before baseline measurements, all specimens were stored in freshly prepared artificial saliva at 37°C for 24 h to allow initial water sorption and postpolymerization stabilization of the composite material, thereby ensuring more clinically relevant baseline conditions.

The artificial saliva used as the control medium was prepared according to ISO 11143:2014 specifications and consisted of potassium phosphate (0.4 g/L), sodium chloride (0.4 g/L), sodium phosphate (0.3 g/L), and sodium carboxymethylcellulose (1.0 g/L) in distilled water with a pH of 6.75 ± 0.05.

### 2.2. Experimental Groups and Immersion Protocol

Based on the immersion media, 40 specimens were divided into four groups (*n* = 10 each) at random.

• Group AS (control): artificial saliva
• Group KC: kombucha (commercially available, pH 2.8 ± 0.2)
• Group AC: apple cider (commercially available, pH 3.5 ± 0.2)
• Group WW: white wine (commercially available, pH 3.2 ± 0.2)

To replicate extended beverage exposure patterns compatible with dietary consumption, each specimen was submerged every day for 24 h in 10 mL of the designated solution kept at 37°C. Following each daily immersion cycle, specimens were carefully wiped with absorbent tissue paper, properly rinsed with distilled water to eliminate any remaining immersion medium, and kept in freshly made artificial saliva at 37°C until the next immersion cycle. This immersion protocol was adapted from previously published methodologies evaluating beverage‐induced staining of dental composite materials and provides temporal and thermal conditions approximating oral environments.

Measurements of color and surface roughness were conducted at baseline (following 24‐h conditioning in artificial saliva), at 7 and 14 days of immersion for color assessment, and at 28 days for surface roughness evaluation. All measurements were performed with specimens in a standardized hydrated state after removal from the immersion medium.

### 2.3. Color Stability Assessment

A spectrophotometer (VITA Easyshade; VITA Zahnfabrik, Bad Sơingen, Germany) was used to obtain colorimetric measurements using Commission Internationale de l’Éclairage (CIE) LAB color space coordinates. *L* ^∗^ stands for lightness (0 = black and 100 = white), *a* ^∗^ for location on the red–green axis (negative = green and positive = red), and *b* ^∗^ for position on the yellow–blue axis (negative = blue and positive = yellow). Before every measurement session, the spectrophotometer was calibrated using a white reference standard in accordance with the manufacturer’s instructions. All measurements were performed by a single calibrated operator to eliminate interoperator variability. To ensure consistency and reproducibility, the spectrophotometer probe was positioned perpendicular to the center of each specimen using a standardized placement protocol. Three consecutive readings were recorded for each specimen at each time point, and mean values were used for analysis.

Color coordinates were measured at four time points: (1) baseline, after specimen conditioning in artificial saliva for 24 h, (2) after 7 days of immersion (*t*_1_), and (3) after 14 days of immersion (*t*_2_). Specimens were washed with distilled water and lightly wiped with absorbent tissue paper to eliminate surface moisture before each color measurement. Each specimen had three duplicate color measurements taken at each time point, and statistical analysis was performed using the mean values.

The CIE LAB 1976 formula was used to determine the overall color change (Δ*E*):

ΔE=ΔL∗222+Δa∗+Δb∗.

### 2.4. Surface Roughness Measurement

A three‐dimensional noncontact optical profilometer was used to measure surface roughness characteristics (ST400; NANOVEA, Micro Photonics Inc., Allentown, PA, USA). This instrument utilizes white light interferometry to capture complete three‐dimensional surface topography without contact with the specimen surface, thereby eliminating potential damage to the composite resin surface during measurement. Each specimen underwent a single standardized scan, capturing the complete polished surface area with automatic computation of roughness parameters.

Surface roughness measurements evaluated multiple ISO 25178‐2:2012‐defined parameters including Sz, the entire surface height from lowest valley to highest peak; Sp, the maximum peak height; Sv, the maximum valley depth; and Ssk, the Ssk, which describes the asymmetry of surface height distribution and indicates whether the surface is dominated by peaks or valleys.

Measurements were recorded at baseline (immediately following polishing and 24‐h conditioning) and at 28 days of daily immersion cycles. Three separate measurement locations on each specimen were evaluated, and mean values were calculated for statistical analysis.

The choice of 28 days for surface roughness measurement reflects the cumulative degradation expected from 28 daily immersion‐rinsing cycles and permits comprehensive assessment of material performance under extended exposure conditions.

### 2.5. Statistical Analysis

IBM SPSS Statistics version 27 (IBM Corp., Armonk, NY, USA) was used for all statistical analyses. For all color parameters (*L* ^∗^, *a* ^∗^, *b* ^∗^, and Δ*E*) and surface roughness variables (Sp, Sv, Sz, and Ssk), descriptive statistics such as means and standard deviations were computed. The Shapiro–Wilk test was used to determine whether the data distribution was normal, and Levene’s test was used to determine whether the variances were homogeneous.

One‐way analysis of variance (ANOVA) with Tukey’s honestly significant difference (HSD) post hoc test was used to compare the four immersion groups at each time point (7, 14, and 28 days) in order to find pairwise differences between groups. Color measurements between the 7‐day and 14‐day time points within each treatment group were compared using paired *t*‐tests for within‐group temporal changes. Because some roughness metrics showed non‐normal distributions, Kruskal–Wallis nonparametric testing was used for surface roughness comparisons. A threshold value of *p* < 0.05 was used to determine statistical significance.

To measure the degree of differences between groups, effect sizes were computed using eta‐squared (*η* ^2^) for ANOVA; a large effect was defined as *η* ^2^ > 0.14.

## 3. Results

### 3.1. Color Stability Findings

One‐way ANOVA showed statistically significant differences between all four groups for all evaluated color parameters (*L* ^∗^, *a* ^∗^, *b* ^∗^, and Δ*E*; *p* < 0.001) at the 7‐day evaluation point (Table 1). The kombucha group showed the lowest *L* ^∗^ values, suggesting more darkening, while the artificial saliva control group showed the highest *L* ^∗^ values, indicating optimal brightness. The artificial saliva group showed minimal progressive increases in *a* ^∗^ and *b* ^∗^ coordinates, while the apple cider and white wine groups demonstrated moderate increases. The kombucha group showed the most substantial changes in both parameters, with the largest overall color shift (Δ*E* = 11.69 ± 1.47 Δ*E* units), significantly exceeding the clinical threshold of Δ*E* = 3.3 units, below which color variations are undetectable to the human eye.

**Table 1:** Color change (Δ*E*) at 7 and 14 days.

Beverage group	Δ*E* at 7 days ± SD	Δ*E* at 14 days ± SD	Change 7→14	vs. Control (14 days)	*p*‐Value
Artificial saliva (control)	0.43 ± 0.31	1.29 ± 0.79	+0.86 (NS)	Baseline	—
Kombucha	11.69 ± 1.47	14.04 ± 1.59	+2.35 ^∗∗∗^	10.9×	<0.001
Apple cider	4.33 ± 0.92	5.63 ± 1.15	+1.30 ^∗∗∗^	4.4×	<0.001
White wine	6.26 ± 1.01	7.96 ± 1.06	+1.70 ^∗∗∗^	6.2×	<0.001

*Note:* Clinical threshold: Δ*E* = 3.3. All beverage groups exceeded threshold by 7 days and showed significant temporal increase (*p* < 0.001), while control remained stable (*p* = 0.148).

Abbreviation: NS, not significant (*p* > 0.05).

^∗∗∗^ *p* < 0.001.

**Figure 1:**
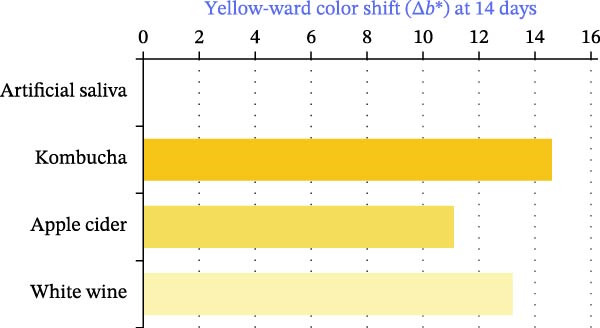
Yellow‐Ward Color Shift (Δ*b*) at 14 Days. Bar graph showing the yellow‐ward color shift (Δ*b*  ^∗^) for each immersion group after 14 days. Kombucha demonstrated the highest shift (~14.6), followed by White Wine (~13.2), Apple Cider (~11.1), and the Artificial Saliva control (~0). The increased Δ*b*  ^∗^ values indicate progressive yellowing of the specimens, with kombucha producing the most pronounced effect.

Figure 1 (color change over time) demonstrates a progressive color change from 7 to 14 days across all beverage groups. The kombucha group (red line) showed the highest color change at 7 days (11.69 ± 1.47 Δ*E* units, Table 1), continuing to progress at 14 days (14.04 ± 1.59 Δ*E* units, Table 1). All beverage groups exceeded the clinically perceptible threshold of Δ*E* = 3.3 by day 7, indicating that color changes were visible to the human eye within the first week of exposure.

**Figure 2:**
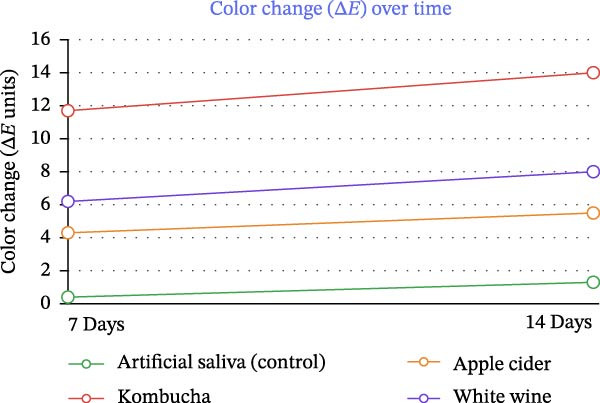
Color change (Δ*E*) Over Time. Line graph showing progressive color change (Δ*E*) from 7 to 14 days across all immersion groups. Kombucha (red) demonstrated the highest color change at both timepoints (11.69 → 14.04 Δ*E* units), followed by White Wine (6.26 → 7.96), Apple Cider (4.33 → 5.63), and the Artificial Saliva control (0.43 → 1.29). All three beverage groups exceeded the clinically perceptible threshold (Δ*E* = 3.3) by day 7, indicating visually detectable discoloration within the first week of exposure, with staining increasing progressively over time.

Following 14 days of continuous immersion, statistically significant differences persisted among all groups for all color parameters (*p* < 0.001, Figure 2 and Table 1). The artificial saliva control group continued to demonstrate minimal discoloration (Δ*E* = 1.29 ± 0.79 units), while Δ*E* values increased substantially in all test beverage groups. The color change ranking at 14 days was Kombucha (14.04) > white wine (7.96) > apple cider (5.63) > artificial saliva (1.29) (Table 1). All test beverage groups exceeded the clinically perceptible threshold by 14 days, definitively indicating that color changes were observable to patients and would require clinical intervention consideration.

Intragroup analysis using paired *t*‐tests comparing color measurements between 7 and 14 days (Table 1) demonstrated significant temporal increases in color change for all beverage groups. Kombucha showed the largest temporal increase (Δ*t*_7–14_ = 2.35 Δ*E* units, *p* < 0.001), followed by white wine (Δ*t*_7–14_ = 1.70 Δ*E* units, *p* < 0.001) and apple cider (Δ*t*_7–14_ = 1.30 Δ*E* units, *p* < 0.001). In marked contrast, the artificial saliva control group did not exhibit a statistically significant change in Δ*E* values between 7 and 14 days (*p* = 0.148), indicating robust color stability in the absence of chromogenic exposure. This temporal pattern demonstrates progressive, cumulative staining that accelerates over time with continued beverage exposure.

**Figure 3:**
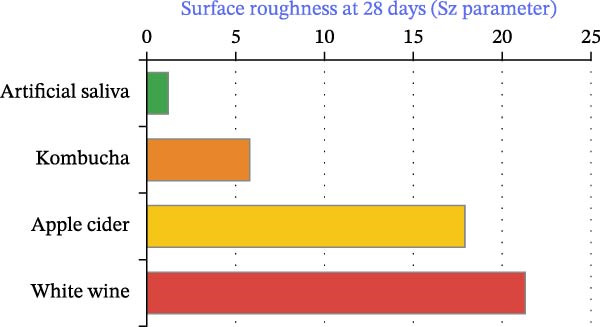
Surface Roughness at 28 Days (Sz Parameter). Bar graph comparing total surface height (Sz, µm) across immersion groups after 28 days. White Wine produced the greatest surface roughness (21.31 µm), followed by Apple Cider (17.93 µm), Kombucha (5.85 µm), and the Artificial Saliva control (1.26 µm). Sz is highlighted as the most clinically relevant measure of surface erosion severity and plaque‐retention risk. Intergroup differences were statistically significant (Kruskal–Wallis, *p* = 0.016).

**Figure 4:**
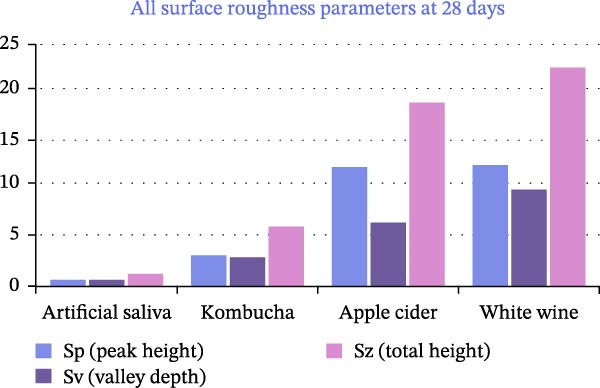
All surface roughness parameters at 28 Days. Grouped bar graph comparing maximum peak height (Sp), maximum valley depth (Sv), and total surface height (Sz) (all in µm) across immersion groups after 28 days. All three parameters increased progressively with beverage treatment, with White Wine (Sp = 11.85, Sv = 9.38, Sz = 21.31) and Apple Cider (Sp = 11.64, Sv = 6.22, Sz = 17.93) showing the most pronounced surface degradation, Kombucha showing moderate alterations (Sp = 3.01, Sv = 2.84, Sz = 5.85), and the Artificial Saliva control remaining lowest (Sp = 0.62, Sv = 0.63, Sz = 1.26).

Analysis of CIE LAB coordinates (Figure 1 and Table 2) revealed predominantly positive *Δ* *b* ^∗^ values in all beverage groups, indicating a shift toward the yellow spectrum and reflecting yellow‐ward discoloration of the composite material. The magnitude of *Δ* *b* ^∗^ values ranged from 11.16 ± 0.36 (apple cider) to 14.69 ± 0.66 (kombucha) at 14 days (Table 2), with kombucha showing the most pronounced yellow shift, suggesting common mechanisms involving yellow‐pigment adsorption or intrinsic matrix yellowing despite the different chemical compositions of the test beverages. Changes in *Δ* *a* ^∗^ (red–green axis) and *Δ* *L* ^∗^ (lightness) were considerably smaller and less consistent across groups (Figures 3 and 4), indicating that the yellow‐ward shift is the predominant color change characteristic across all beverages.

**Table 2:** CIE LAB color coordinates and yellow‐ward shift at 14 days.

Beverage	*Δ* *L* ^∗^ (lightness)	*Δ* *a* ^∗^ (red–green)	*Δ* *b* ^∗^ (yellow) ± SD	*Δ* *b* ^∗^ magnitude	Primary color mechanism
Artificial saliva	Minimal	Minimal	Minimal	Control	Stable
Kombucha	Darkening	Sig. red shift	14.69 ± 0.66	Highest	→Yellow shift
Apple cider	Moderate ↓	Moderate red	11.16 ± 0.36	Lowest	→Yellow shift
White wine	Moderate ↓	Moderate red	13.24 ± 0.48	High	→Yellow shift

*Note:* All *Δ* *b* ^∗^ values are positive, indicating yellow‐ward discoloration as a common staining mechanism. ↓ indicates a decrease in lightness. Kombucha shows additional darkening (*Δ* *L* ^∗^) and reddening (*Δ* *a* ^∗^) contributing to severe overall color change.

### 3.2. Surface Roughness Alterations at 28 Days

Surface roughness characteristics of the single‐shade composite resin were evaluated after 28 days of immersion using three‐dimensional optical profilometry. The surface topography was quantified using ISO 25178 parameters, including maximum peak height (Sp), maximum valley depth (Sv), total surface height (Sz), and Ssk. Descriptive statistics of all surface roughness parameters at 28 days are presented in Table 3. Figure 4 displays grouped bar charts comparing all three major roughness parameters (Sp, Sv, and Sz) across all groups, demonstrating a clear dose–response relationship with beverage treatment.

**Table 3:** Surface roughness parameters (Sp, Sv, and Sz) at 28 days.

Beverage	Sp (µm)	Sp fold	Sv (µm)	Sv fold	Sz (µm)	Sz fold	Clinical severity
Artificial saliva	0.62 ± 0.002	1.0×	0.63 ± 0.002	1.0×	1.26 ± 0.005	1.0×	Minimal/stable
Kombucha	3.01 ± 0.05	4.9×	2.84 ± 0.03	4.5×	5.85 ± 0.09	4.6×	Moderate deg.
Apple cider	11.64 ± 0.04	18.8×	6.22 ± 0.06	9.9×	17.93 ± 0.04	14.3×	High deg.
White wine	11.85 ± 0.06	19.1×	9.38 ± 0.20	14.9×	21.31 ± 0.20	16.9×	Severe deg.

*Note:* Sp, maximum peak height; Sv, maximum valley depth; Sz, total surface height (most clinically relevant for plaque retention, threshold ~0.2 µm). All beverages substantially exceed critical threshold. Kruskal–Wallis test: *p* = 0.016 (significant). White wine produces a 16.9× increase in the Sz parameter.

The artificial saliva group exhibited the lowest values for all roughness parameters: Sp (0.62 ± 0.002 µm), Sv (0.63 ± 0.002 µm), and Sz (1.26 ± 0.005 µm) (Table 3). These minimal values confirm the stability of the polished composite surface in the absence of acidic or erosive challenges and validate artificial saliva as a suitable control medium that does not induce iatrogenic surface degradation.

The kombucha group demonstrated moderate increases in surface roughness parameters (Table 3), with mean values of Sp = 3.01 ± 0.05 µm, Sv = 2.84 ± 0.03 µm, and Sz = 5.85 ± 0.09 µm. These findings suggest measurable surface degradation representing a 4.6‐fold increase over control for the Sz parameter (Table 3), likely related to the acidic nature of kombucha (pH 2.8 ± 0.2) and its fermentation by‐products, which can induce superficial resin matrix softening and modest filler‐matrix debonding without causing the extensive erosive damage observed with other beverages.

Apple cider immersion resulted in substantially higher roughness values (Table 3), with Sp reaching 11.64 ± 0.04 µm (18.8 × control), Sv = 6.22 ± 0.06 µm (9.9 × control), and Sz = 17.93 ± 0.04 µm (14.3 × control, Table 3). The pronounced increase in these parameters reflects significant surface degradation, which may be attributed to the acidic pH of apple cider (3.5 ± 0.2) combined with the presence of organic acids such as malic acid. Prolonged exposure to such acidic environments weakens the filler‐matrix interface, promotes filler particle loss, and increases surface irregularities and topographical variation.

The white wine group exhibited the greatest surface roughness among all tested beverages (Table 3). White wine produced Sp = 11.85 ± 0.06 µm (19.1 × control), Sv = 9.38 ± 0.20 µm (14.9 × control), and most notably, Sz = 21.31 ± 0.20 µm (16.9 × control), values substantially exceeding those of all other groups. This pronounced surface alteration can be explained by the combined effect of low pH (3.2 ± 0.2) and the ethanol content in white wine. Ethanol acts as a plasticizer of the resin matrix, enhancing polymer chain mobility, promoting matrix softening, and facilitating filler‐matrix debonding, while organic acids (tartaric, malic, and citric acids) simultaneously promote hydrolytic breakdown of ester linkages within the polymer structure, further contributing to erosive surface degradation and topographical irregularities.

Ssk values also differed significantly among the groups (Table 3), indicating variations in the surface feature distribution. The artificial saliva group showed the highest Ssk value, suggesting a more uniform and symmetrical surface profile. In contrast, lower Ssk values observed in all beverage groups reflect increased surface irregularity with a predominance of deeper valleys and sharper peaks following acidic exposure, creating a more hostile surface topography for oral tissues.

Figure 3 (surface roughness at 28 days–Sz parameter) highlights the Sz parameter specifically, as it represents the most clinically relevant measure of surface erosion severity and plaque retention risk (critical threshold ~0.2 µm). The roughness ranking from highest to lowest was as follows: white wine (21.31 µm, red bar) > apple cider (17.93 µm, yellow bar) > kombucha (5.85 µm, orange bar) > artificial saliva (1.26 µm, green bar).

Intergroup comparison using the Kruskal–Wallis nonparametric test (appropriate due to non‐normal distribution of some roughness variables) revealed statistically significant differences among the four immersion media for all evaluated surface roughness parameters (Sp, Sv, Sz, and Ssk) (*p* = 0.016, Table 4). Therefore, the null hypothesis that there are no differences in surface roughness among the groups was definitively rejected, confirming that beverage immersion produces measurable and statistically significant surface topographical alterations.

**Table 4:** Statistical analysis summary.

Analysis type	Statistical test	Result	Significance
Color change (7 and 14 days)	One‐way ANOVA	*p* < 0.001	^∗∗∗^Highly significant
Pairwise comparisons	Tukey post hoc	All vs. control	^∗∗∗^ *p* < 0.001
Surface roughness (28 days)	Kruskal–Wallis	*p* = 0.016	^∗^Significant
Temporal changes (7→14 days)	Paired *t*‐test	Beverages: *p* < 0.001	^∗∗∗^Significant
Control stability (7→14 days)	Paired *t*‐test	*p* = 0.148	Not significant
Effect size	Eta‐squared (*η* ^2^)	*η* ^2^ > 0.14	Large effect

*Note:* All beverage groups showed statistically significant color changes and roughness alterations compared to control. Control group demonstrated remarkable stability, validating artificial saliva as an appropriate control medium.

^∗^ *p* < 0.05 = significant.

^∗∗^ *p* < 0.01 = highly significant.

^∗∗∗^ *p* < 0.001 = very highly significant.

### 3.3. Critical Dissociation Between Color Change and Surface Roughness

A notable and clinically significant finding emerged from the comparative analysis of color change and surface roughness severity. Despite kombucha demonstrating the highest color change severity (Δ*E* = 14.04 at 14 days, ranking 1st worst in discoloration), it produced only moderate surface roughness alterations (Sz = 5.85 µm, ranking 3rd). Conversely, white wine produced the most severe surface roughness degradation (Sz = 21.31 µm, ranking 1st worst in roughness) but generated intermediate‐level discoloration (Δ*E* = 7.96 at 14 days, ranking 2nd).

Overall, the results comprehensively demonstrate that acidic and chromogenic beverages induce significant topographical changes in single‐shade composite resin, with the severity of roughness alterations following the order: white wine (Sz = 21.31 µm) > apple cider (Sz = 17.93 µm) > kombucha (Sz = 5.85 µm) > artificial saliva (Sz = 1.26 µm), while color change severity follows the inverse ranking: kombucha (Δ*E* = 14.04) > white wine (Δ*E* = 7.96) > apple cider (Δ*E* = 5.63) > artificial saliva (Δ*E* = 1.29). This dissociation between color and roughness severity has significant clinical implications for patient counseling and preventive strategies, requiring beverage‐specific management approaches tailored to the primary risk profile (esthetic vs. functional) of each beverage type.

## 4. Discussion

The current study’s findings show that single‐shade composite resin undergoes measurable and clinically significant changes in both color stability and surface roughness when exposed to acidic and chromogenic beverages commonly consumed in contemporary diets. These findings extend previous research on traditional multishade composites by specifically documenting the vulnerability of single‐shade materials to extrinsic staining and surface degradation.

The predominance of yellow‐ward color shifts (positive *Δ* *b* ^∗^ values) observed across all beverage groups suggests common staining mechanisms involving either extrinsic pigment adsorption or intrinsic matrix yellowing. This yellow‐ward shift may be attributed to chromogenic compounds such as polyphenols and tannins present in beverages like kombucha and apple cider, which can adsorb onto or diffuse into the resin matrix. Kombucha induced the greatest color change, which may be explained by its high concentration of tannins, polyphenolic chemicals, and fermentation by‐products derived from its tea base. These chromogenic molecules possess multiple aromatic rings that facilitate strong interactions with the composite resin surface through both physical adsorption and potential diffusion into the less densely cross‐linked superficial regions of the matrix [27, 28].

In contrast to color changes, white wine produced the greatest alteration in surface roughness parameters despite generating intermediate‐level discoloration. This dissociation between staining intensity and surface roughness enhancement suggests distinct degradation mechanisms. Ssk values also differed significantly among the groups, indicating variations in surface feature distribution. The artificial saliva group showed higher Ssk values, suggesting a more uniform surface profile, whereas reduced Ssk values observed in beverage‐treated groups indicate a surface increasingly dominated by valleys rather than peaks. This shift in surface morphology may enhance plaque retention, staining susceptibility, and overall surface degradation. The chemical composition of white wine—including low pH (3.2), ethanol content, and organic acids (tartaric, malic, and citric)—facilitates multifaceted resin matrix degradation. Ethanol acts as a solvent for the organic polymer matrix, enhancing molecular mobility and facilitating matrix plasticization and softening. Simultaneously, organic acids promote the hydrolytic breakdown of ester linkages within the dimethacrylate resin polymer, weakening intermolecular bonds [29, 30]. These combined effects accelerate filler‐matrix debonding and progressive surface erosion, resulting in visible topographical irregularities [30, 31].

Apple cider exhibited an intermediate degradation profile, with moderate discoloration and surface roughness alterations. Although apple cider possesses a lower pH than white wine (pH 3.5 vs. 3.2), it lacks the plasticizing effect of ethanol, resulting in less severe surface erosion. Discoloration associated with apple cider is likely attributable to natural pigments and phenolic compounds present in apple juice that adsorb onto or diffuse into the resin matrix surface [32].

Kombucha presented a unique profile characterized by pronounced discoloration despite comparatively lower surface roughness alterations relative to white and apple cider. This pattern indicates that kombucha’s staining potential derives primarily from its high chromogenic content rather than its erosive capability. The fermented tea base provides abundant tannins and polyphenols that readily penetrate and discolor the composite surface without substantially degrading the underlying polymer matrix structure [33].

The artificial saliva control group demonstrated remarkable color and surface stability, undergoing minimal measurable changes throughout the 28‐day investigation. This observation validates the use of artificial saliva as an appropriate neutral control medium and supports the contention that observed changes in beverage‐immersed groups result from the specific chemical interactions between the test solutions and the composite resin material. The slight color and roughness variations detected in controls likely reflect normal water sorption and hygroscopic expansion of the resin matrix, processes that are significantly amplified by the acidic and chromogenic challenge of the test beverages.

The temporal increase in color change observed between 7 and 14 days (ranging from 1.3 to 3.3 Δ*E* units depending on the beverage) indicates cumulative staining with prolonged exposure. This temporal pattern has significant clinical implications, suggesting that patients consuming these beverages regularly will experience progressive discoloration over time, potentially reaching clinically unacceptable levels within 2–4 weeks of typical consumption patterns. In contrast, the minimal temporal changes observed in the control group underscore the protective effect of a neutral aqueous environment.

The relationship between surface roughness and color stability warrants further discussion. Kombucha induced high discoloration without proportional surface roughness increases, suggesting that surface irregularities alone do not fully explain staining vulnerability. Instead, both the intrinsic material properties (hydrophilicity, cross‐link density, and filler‐matrix bonding quality) and the specific chemical nature of staining agents play important roles. High‐polarity chromogenic compounds in kombucha may penetrate smooth surfaces through diffusion, while more physically dependent staining mechanisms (pigment trapping in surface irregularities) predominate with solutions having greater erosive potential.

From a clinical perspective, these findings suggest that frequent consumption of acidic and chromogenic beverages may compromise both the esthetic appearance and surface integrity of composite restorations within a relatively short period. The observed dissociation between color change and surface degradation further indicates that different beverages may pose distinct risks, requiring tailored preventive strategies. Clinicians should therefore emphasize dietary counseling, recommend minimizing direct contact through the use of straws, and advise rinsing after beverage consumption. Periodic polishing and maintenance may also help preserve restoration longevity and esthetic outcomes [34, 35].

Limitations of the present study warrant acknowledgment. This investigation employed only one single‐shade composite resin material (Omnichroma), and the results may not be generalizable to other single‐shade products with different filler technologies or matrix chemistries. Additionally, the continuous immersion protocol may not fully replicate the intermittent exposure patterns of beverage consumption in clinical settings. The 28‐day duration is relatively short compared to the expected 5–10‐year clinical lifespan of composite restorations; longer‐term investigations are needed to determine whether the observed trends continue or plateau. Furthermore, thermocycling was not employed in this investigation, and temperature fluctuations are known to influence material degradation and water sorption. Finally, this study evaluated only three beverages, and inclusion of additional contemporary beverages (e.g., energy drinks, plant‐based beverages, and specialty coffee preparations) would provide more comprehensive information regarding dietary risk factors.

Future investigations should employ multiple single‐shade composite materials to determine whether the observed effects are material‐specific, utilize longer immersion durations combined with thermocycling and mechanical stressing to more closely simulate oral conditions, and evaluate the efficacy of preventive interventions such as surface sealants or protective coatings in mitigating beverage‐induced degradation. Additionally, clinical studies documenting the actual performance of single‐shade composites in longitudinal follow‐up would strengthen the understanding of in vivo material behavior.

## 5. Conclusions

Single‐shade composite resin (Omnichroma) showed considerable susceptibility to extrinsic discoloration and surface degradation when exposed to acidic and chromogenic beverages under the constraints of this in vitro study. Kombucha induced the greatest color changes, likely due to high polyphenol and tannin contents, while white wine produced the most pronounced surface roughness alterations through combined ethanol and organic acid degradation mechanisms. The dissociation between staining intensity and surface roughness changes indicates multifactorial degradation pathways operating simultaneously.

These results emphasize how crucial patient education and preventative measures are to maximizing the clinical efficacy of single‐shade composite restorations. Clinicians should discuss dietary habits with patients and emphasize the necessity of limiting exposure to chromogenic and acidic beverages, employing protective strategies such as straws and prompt rinsing, and attending regular preventive appointments for professional surface maintenance. Single‐shade composites remain clinically valuable materials, but their use should be accompanied by realistic patient expectations regarding esthetic stability and appropriate dietary counseling. Future research employing multiple materials, extended immersion durations, and thermomechanical cycling will provide a more comprehensive understanding of single‐shade composite durability in the oral environment.

## Funding

This research received no specific grant from any funding agency in the public, commercial, or not‐for‐profit sectors.

## Conflicts of Interest

The authors declare no conflicts of interest.

## Data Availability

The data supporting the findings of this study are available from the corresponding author upon reasonable request.
